# Physiological and Neural Adaptations to Eccentric Exercise: Mechanisms and Considerations for Training

**DOI:** 10.1155/2015/193741

**Published:** 2015-10-12

**Authors:** Nosratollah Hedayatpour, Deborah Falla

**Affiliations:** ^1^Center for Biomechanic and Motor Control (BMC), Department of Physical Education and Sport Science, University of Bojnord, Bojnord, Iran; ^2^Department of Neurorehabilitation Engineering, Bernstein Center for Computational Neuroscience, University Medical Center Göttingen, Georg-August University, 37075 Göttingen, Germany; ^3^Pain Clinic, Center for Anesthesiology, Emergency and Intensive Care Medicine, University Hospital Göttingen, 37075 Göttingen, Germany

## Abstract

Eccentric exercise is characterized by initial unfavorable effects such as subcellular muscle damage, pain, reduced fiber excitability, and initial muscle weakness. However, stretch combined with overload, as in eccentric contractions, is an effective stimulus for inducing physiological and neural adaptations to training. Eccentric exercise-induced adaptations include muscle hypertrophy, increased cortical activity, and changes in motor unit behavior, all of which contribute to improved muscle function. In this brief review, neuromuscular adaptations to different forms of exercise are reviewed, the positive training effects of eccentric exercise are presented, and the implications for training are considered.

## 1. Introduction

Neuromuscular and functional changes induced by exercise are specific to the mode of exercise performed. The degree of mechanical tension, subcellular damage, and metabolic stress can all play a role in exercise-induced muscle adaptations [1–5]. Of the three types of muscle contractions that can be utilized during exercise (concentric, isometric, and eccentric), eccentric exercises are those actions in which the muscle lengthens under tension. During eccentric contractions the load on the muscle is greater than the force developed by the muscle and the muscle is stretched, producing a lengthening contraction. Eccentric exercise is characterized by muscle microlesions and greater mechanical tension as compared to concentric/isometric contractions and therefore may result in greater muscle adaptations. Although all forms of exercise may induce impressive muscle adaptation, it is not always clear which method is best for maximizing adaptation gains. This paper provides a brief overview of studies documenting physiological (metabolic, histochemical) and neural adaptations in response to exercise training, with an emphasis on eccentric exercise.

## 2. Exercise Training and Physiological Adaptations

High intensity resistance training is associated with significant physiological adaptations within skeletal muscle [6] including changes in the contractile and/or noncontractile elements of muscle. When mechanical overload of muscle occurs, the myofibers and extracellular matrix are disturbed, which in turn stimulates a process of protein synthesis [7]. Mechanical tension induced by high intensity exercise can also increase the rate of metabolic stress and stimulate subcellular pathways involved in protein synthesis such as the mitogen-activated protein kinase pathway, which may play a role in exercise-induced muscle growth [1, 2]. The total number of sarcomeres in parallel and in series increase resulting in an increase in fascicle length and pennation angle and, consequently, muscle hypertrophy. It has been proposed that stretch combined with overload is the most effective stimulus for promoting muscle growth [8, 9]. During eccentric exercise, skeletal muscle is subjected to both stretch and overload which triggers subcellular damage to the contractile and structural components of skeletal muscle [10, 11]. This subcellular damage induces a sequence of physiological events including the activation of master signaling pathways for gene expression and muscle hypertrophy [1, 8, 10]. Notwithstanding, mechanotransduction (exercise-induced mechanical stimuli) may be the primary mechanism associated with muscle hypertrophy in healthy muscle. This is demonstrated by an increase in the number of sarcomeres in the absence of fiber necrosis following exercise-induced muscle tension [12]. Skeletal muscles sense mechanical information and convert this stimulus into the biochemical events that regulate the rate of protein synthesis. However, since eccentric contractions induce greater mechanical tension on the muscle fibers than concentric exercise, this form of exercise induces a more rapid addition of sarcomeres in series and in parallel as inferred from the increase in muscle cross sectional area (CSA) and pennation angle [13]. Previous studies reported an increase of fiber length in muscles subjected to chronic eccentric work [14], whereas a decrease [14] or a lack of change [15] of fiber length was shown in muscles worked concentrically. Greater muscle hypertrophy following high intensity eccentric exercise was also associated with larger fiber pennation angle [15]. These results indicate that the mechanical stimuli induced by high intensity exercise may be a primary mechanism for muscle hypertrophy. Hortobágyi et al. [16] also observed that muscle mass recovery after immobilization was greatest following eccentric exercise compared to concentric and isometric training, most likely due to the greater mechanical tension produced during eccentric exercise [17]. Similarly, other studies demonstrated that high tension eccentric exercise is more effective than concentric exercise in increasing muscle mass, through changes in histochemical characteristics and metabolic substrates within the skeletal muscle [18].

### 2.1. Eccentric Exercise and Histochemical Adaptations

The mechanisms underlying the hypertrophic response to exercise may include changes in the hormonal milieu, cell swelling, free-radical production, and increased activity of growth-oriented transcription factors [6, 7]. Mechanical tension, produced by force generation and stretch, is an essential factor to stimulate signaling pathways involved in muscle growth, and the combination of these stimuli appears to have a marked additive effect [9, 19, 20]. Mechanical stimuli can regulate the rate of protein synthesis through changes in binding of a ribosome to the mRNA and/or by modifications in methylguanosine, which in turn encodes proteins that are central to the growth process [21]. Mechanical stimuli may also contribute to muscle hypertrophy through changes in muscle fiber membrane permeability to calcium ions [22]. The increased calcium concentrations within the cytosol of the muscle cell increase the rate of protein synthesis in skeletal muscle [23]. Moreover, titin is a site for calcium binding and is ideally positioned in the muscle sarcomere to sense mechanical stimuli and transform them into biochemical signals, capable of altering sarcomere number and optimal tension during lengthening contractions [24, 25].

During eccentric exercise the contracting muscle is forcibly stretched, producing a higher mechanical tension and muscle microlesions. Mitogen-activated protein kinase is a master signaling pathway for gene expression and muscle hypertrophy [26] and is considered to be the most responsive to mechanical tension and subcellular muscle damage [1]. Mitogen-activated protein kinase links cellular stress with an adaptive response in myocytes, modifying growth and differentiation [7, 27]. Insulin-like growth factor is also considered to be a key factor for muscle hypertrophy and shows enhanced effects in response to mechanical loading [28, 29]. Insulin-like growth factor contributes to muscle hypertrophy through a mechanical response of IGF-1Ea isoform to exercise training and appears to be activated by mechanical signals and subcellular muscle damage [28, 30]. Mechanical stimulation may cause the IGF-1 gene to be spliced toward IGF-1Ea isoform which in turn increases IGF-IEa mRNA expression [31] and muscle hypertrophy [32].

Muscle hypertrophy following eccentric exercise may also be explained by other tension-sensitive anabolic pathways. For example, the effects of testosterone on muscle hypertrophy are enhanced by mechanical loading, either directly by increasing the rate of protein synthesis and inhibiting protein breakdown [33] and/or indirectly by stimulating the release of other anabolic hormones such as Growth hormone [34]. Bamman et al. [35] reported that high intensity eccentric exercise upregulated androgen receptor content in humans and modulation of androgen receptor content appears to occur predominantly in fast-twitch muscle fibers [36]. Accordingly, Ahtiainen et al. [37] reported significant correlations between training intensity, testosterone concentration, and muscle cross-sectional area, indicating that high intensity eccentric exercise-induced elevation in testosterone is an important contributor to muscle hypertrophy.

Growth hormone may contribute to muscle hypertrophy through both anabolic and catabolic processes. An increase in Growth hormone can enhance interaction with muscle cell receptors, facilitating fiber recovery and stimulating a hypertrophic response [38]. Other anabolic signaling pathways including calcium-dependent pathways have been implicated in the regulation of muscle hypertrophy [39].

### 2.2. Eccentric Exercise and Metabolic Adaptations

Mechanical tension produced by force generation and stretch contributes to muscle ischemia [8, 9] which can lead to metabolic adaptations within the skeletal muscle. During eccentric contractions, passive muscular tension develops because of lengthening of extramyofibrillar elements, especially collagen content in the extracellular matrix which can contribute to an increased acidic environment. Such an environment can contribute to increased fiber degradation and increased sympathetic nerve activity [7], facilitating an adaptive hypertrophic response [2]. Numerous studies indicate that anabolic exercise induced metabolic stress can have a significant hypertrophic effect [2].

## 3. Exercise Training and Neural Adaptations

Neural adaptations to training can be defined as changes within the nervous system that allow a trainee to more fully activate prime movers in specific movements and to better coordinate the activation of all relevant muscles, thereby affecting a greater net force in the intended direction of movement [40]. Neural adaptations may occur at the level of the motor cortex, spinal cord, and/or neuromuscular junction following training [41–43]. Adaptations may also occur at excitation- contraction coupling pathways located distal to the neuromuscular junction. The neural adaptations observed following training explain the disproportionate increase in muscle force compared to muscle size during the initial stages of training. For instance, increased muscle activity, recorded with electromyography (EMG), has been observed during the early phase of strength training in association with significant gains in muscle strength, but in the absence of changes of muscle mass or changes in membrane characteristics within the skeletal muscle [44]. Early gains in strength have been attributed to a variety of mechanisms including increased maximal motor unit discharge rates [45, 46], increased incidence of brief interspike intervals (doublets) [47], and decreased interspike interval variability [48].

Numerous other studies have investigated neural adaptations following resistance training. Aagaard et al. [49] observed increases in evoked V-wave and H-reflex responses during maximal muscle contraction after resistance training indicating an enhanced neural drive in the corticospinal pathways and increased excitability of motor neurons. Furthermore, previous studies have demonstrated significant changes in motor unit discharge rate [46], muscle fiber conduction velocity [50], and rate of force development after resistance training [46, 51]. Collectively these studies show that increased strength following resistance training can be attributed to both supraspinal and spinal adaptations (i.e., increased central motor drive, elevated motoneuron excitability, and reduced presynaptic inhibition) [49].

The neural adaptions to resistance training are dependent on type of muscle contractions performed and the neural adaptations and improvement in muscle force vary depending on whether eccentric, concentric, or isometric contractions are executed [46, 52]. The section below focuses on the specific neural adaptations that have been observed with eccentric exercise.

### 3.1. Eccentric Exercise and Cortical Activity

It is well known that exercise can induce changes in cortical activity [53–55]. These changes can be measured with techniques such as electroencephalography (EEG) and neuroimaging techniques and studies applying these methods have demonstrated that variations in cortical activation patterns depend on exercise mode and intensity [41, 56]. This is perhaps not surprising given that the central nervous system employs a different neural strategy to control skeletal muscle during eccentric contractions versus isometric or concentric muscle contraction. This is evidenced, for example, by the preferential recruitment of fast twitch motor units and different activation levels among synergistic muscles during eccentric compared to concentric contractions [57–59]. Fang et al. [41] showed that cortical activities for movement preparation and execution were greater during eccentric than concentric tasks, most likely due to concurrent modulation (gating by presynaptic input) of the Ia afferent input from the lengthening muscle to reduce the unwanted stretch reflex and subcellular muscle damage [60]. Thus the brain probably plans and programs eccentric movements differently to concentric muscle tasks [41]. Moreover, neuroimaging studies have shown that cortical activities associated with the processing of feedback signals are larger during eccentric than concentric actions, likely due to the higher degree of movement complexity and/or stretch-related transcortical reflexes to control the stretched muscle [61, 62]. Additionally, earlier onset of cortical activation has been observed for eccentric versus concentric contractions [41] which has been attributed to the planning for more movement complexity, modulation of monosynaptic reflex excitability, or carrying out a different control strategy (e.g., motor unit recruitment) for an eccentric action [57, 61, 62].

### 3.2. Eccentric Exercise and Motor Unit Behavior

During a muscle contraction, the central nervous system controls the production of increased muscle force by either increasing motor unit firing rates and/or the recruitment of additional motor units. Numerous studies have investigated changes in motor unit firing rates after resistance training and have shown that the change in motor unit firing rate is dependent on the type of muscle contraction. Van Cutsem et al. [47] observed increased firing rates of motor units and a more frequent occurrence of short interspike intervals (doublets) following 12 weeks of dynamic contractions of the ankle dorsiflexors. Kamen and Knight [63] also found a 15% increase in motor unit firing rates following 6 weeks of dynamic training of the quadriceps muscles. Similarly, Vila-Chã et al. [45] reported a significant increase in firing rates of vasti motor units after six weeks of resistance training. However, other studies have reported no change in maximal motor unit firing rates following isometric resistance training of the abductor digiti minimi and quadriceps muscles despite a significant increase in absolute force [46, 64, 65]. These studies suggest that maximal motor unit firing rates increase in response to dynamic but not isometric resistance training. It has been proposed that stretch combined with overloading is the most effective stimulus for enhancing motor unit firing rates during dynamic resistance exercise. For instance, Dartnall et al. [66] showed ~40% decline in biceps brachii motor unit recruitment thresholds and 11% increase in minimum motor unit discharge rates immediately after and 24 h after eccentric exercise. Thus, more biceps brachii motor units were active at the same relative force after eccentric exercise.

A potential mechanism responsible for the increased muscle activation following eccentric training has been attributed to the neural regulatory pathways involved in the excitation and inhibition process. During eccentric contractions, the spinal inflow from Golgi Ib afferents and joint afferents induce elevated presynaptic inhibition of muscle spindle Ia afferents, as demonstrated by reduced H-reflex responses and EMG amplitude during active eccentric versus concentric contractions [67, 68]. The removal of neural inhibition and the corresponding increase in maximal muscle force and rate of force development observed following eccentric resistance training could be caused by a downregulation of such inhibitory pathways, possibly by central descending pathways [69].

### 3.3. Eccentric Exercise and Muscle Force

Since greater maximum force can be developed during maximal eccentric muscle actions compared to concentric or isometric muscle actions, heavy-resistance training using eccentric muscle actions may be most effective for increasing muscle strength. Eccentric exercise may preferentially recruit fast twitch muscle fibers and perhaps the recruitment of previously inactive motor units [70]. This would lead to increased mechanical tension and as a consequence led to even greater force production [52].

Farthing and Chilibeck [52] reported that 8 weeks of eccentric resistance training resulted in greater muscle hypertrophy and muscle force than training with concentric contractions. In agreement, Kaminski et al. [69] also observed greater improvements in peak torque following eccentric (29%) compared to concentric (19%) training. It has also been shown that ballistic movement with stretch-shortening cycle muscle activation has the greatest effect on enhancing the rate of force development compared to concentric and isometric muscle contractions [71].

## 4. Considerations

Eccentric exercise is characterized by high force generation and low energy expenditure as compared to concentric and isometric exercises [72, 73] and therefore can be beneficial for clinical treatments. For example, eccentric exercise has been used in rehabilitation to manage a host of conditions including rehabilitation of tendinopathies, muscle strains, and anterior cruciate ligament (ACL) injuries [74, 75]. Although there are positive effects of eccentric exercise as reviewed above, it must be noted that there can also be detrimental effects. For instance, the nonuniform effect of eccentric exercise results in nonuniform changes in muscle activation [11], alternative muscle synergies [76] which may lead to strength imbalances. Studies have confirmed that intensive eccentric exercise may have a differential effect on different muscle regions [4, 5, 11, 77, 78] potentially resulting in an imbalance of muscle activity and alteration of the load distribution on joints. Eccentric exercise is also associated with muscle micro lesions, pain, reduced fiber excitability, and initial muscle weakness [4, 77, 79]. Furthermore, eccentric exercise may impair reflex activity which could lead to compromised joint stability during perturbations [43, 80]. Thus it is important to consider the initial unfavorable effects in addition to the long-term benefits.

## 5. Conclusion

Eccentric contractions are important to consider for training and rehabilitation programs because of their potential to produce large force with low metabolic cost. Data reported by several studies suggests that stretch combined with overloading, as in eccentric contractions, is the most effective stimulus for promoting muscle growth and enhancing the neural drive to muscle. This is evidenced by greater muscle hypertrophy, greater neural activity, and larger force production following eccentric exercise versus concentric and isometric exercise. Therefore, training that involves true maximal eccentric loadings could be more effective than concentric and isometric training for developing muscle growth and removing neural inhibition, leading to a significant improvement of muscle function.
